# Seed Set and Natural Regeneration of *Dendrocalamus membranaceus* Munro after Mass and Sporadic Flowering in Yunnan, China

**DOI:** 10.1371/journal.pone.0153845

**Published:** 2016-04-14

**Authors:** Ning Xie, Ling-Na Chen, Khoon-Meng Wong, Yong-Zhong Cui, Han-Qi Yang

**Affiliations:** 1 Research Institute of Resources Insects, Chinese Academy of Forestry, Kunming 650224, Yunnan, People's Republic of China; 2 Singapore Botanic Gardens, 1 Cluny Road, Singapore 259569, Republic of Singapore; Universita degli Studi di Siena, ITALY

## Abstract

The flowering periods of woody bamboos, seed set, natural regeneration and death after flowering have been rarely observed and evaluated in the field. *Dendrocalamus membranaceus* Munro, a tropical woody bamboo mainly distributed in the Yunnan, displayed both sporadic as well as gregarious (mass) flowering and fruited from 2006 to 2013 following severe droughts. The aim of this study is to examine potential differences in seed set and natural regeneration between the two flowering patterns in natural *D*. *membranaceus* forests. We investigated and analyzed seed set, seed germination, seedling growth and mortality in both mass and sporadic flowering populations. Observations were made over a period of three years to record changes in bamboo seedling density, height and culm diameter. We observed a low natural seed set ranging from 1.76% to 7.49%, and a relatively high seed germination rate in the nursery from 59.6% to 71.0% for both types of flowering populations. Seeds germinated in 5–7 days after sowing and the germination period lasted 10–15 days. Seed set and germination rates in mass-flowering populations were significantly higher than those of sporadically flowering stands. The seedlings within sporadically flowering populations died within two years. In comparison, seedling mortality in the mass flowering population increased over two periods of observation from 64.92% to 98.89%, yet there was good seedling establishment left over, which showed mean height and mean culm diameter increasing by 1053.25% and 410.71%, respectively, in the second year of observations, and 137.10%, and 217.48%, respectively, in the third year. There are significant differences in seed set, natural regeneration ability and sustainability of bamboo populations between the mass flowering and sporadically flowering populations of *D*. *membranaceus*. Sporadic flowering populations failed to produce effective regeneration, while mass flowering populations were able to regenerate successfully. This study provides useful insights for conservation and natural forest management of *D*. *membranaceus*. We consider the merits of introducing other genetic provenances towards long-term maintenance of the stand features at sporadically flowering sites; meanwhile, the most economic option for mass flowering stands is to allow natural regeneration to take place through protecting such sites from further disturbance.

## Introduction

*Dendrocalamus membranaceus* Munro is a species of giant woody sympodial bamboo in tropical Asia. The culms of this species have a height of 10–20 m and a diameter of 7–15 cm. It is naturally distributed throughout Myanmar, Laos, north Thailand, northern Vietnam and China’s Yunnan Province [1]. In Yunnan, *D*. *membranaceus* forest covers an area of ca. 7×10^4^ ha, and is mostly distributed in the lower reaches of the Lancang River below an altitude of 1000 m above sea level [2,3,4]. At present, *D*. *membranaceus* forest is the largest and most representative natural stands of tropical large-sized sympodial bamboo found in China [5]. *D*. *membranaceus* is of extensive industrial use, such as for timber, pulp and bamboo shoots [1,4]. Although *D*. *membranaceus* is not a threatened plant on the present Red List of the International Union for Conservation of Nature (IUCN), its main distribution in southern Yunnan includes the Xiaomengyang National Nature Reserve (XNNR) [6], one of the habitats of the wild Asian elephant [7], an endangered species on the IUCN Red List (www.iucnredlist.org). Therefore, *D*. *membranaceus* is a multi-purpose bamboo species not only important for industrial ulitization but also for ecological conservation [1,8].

**Fig 1:**
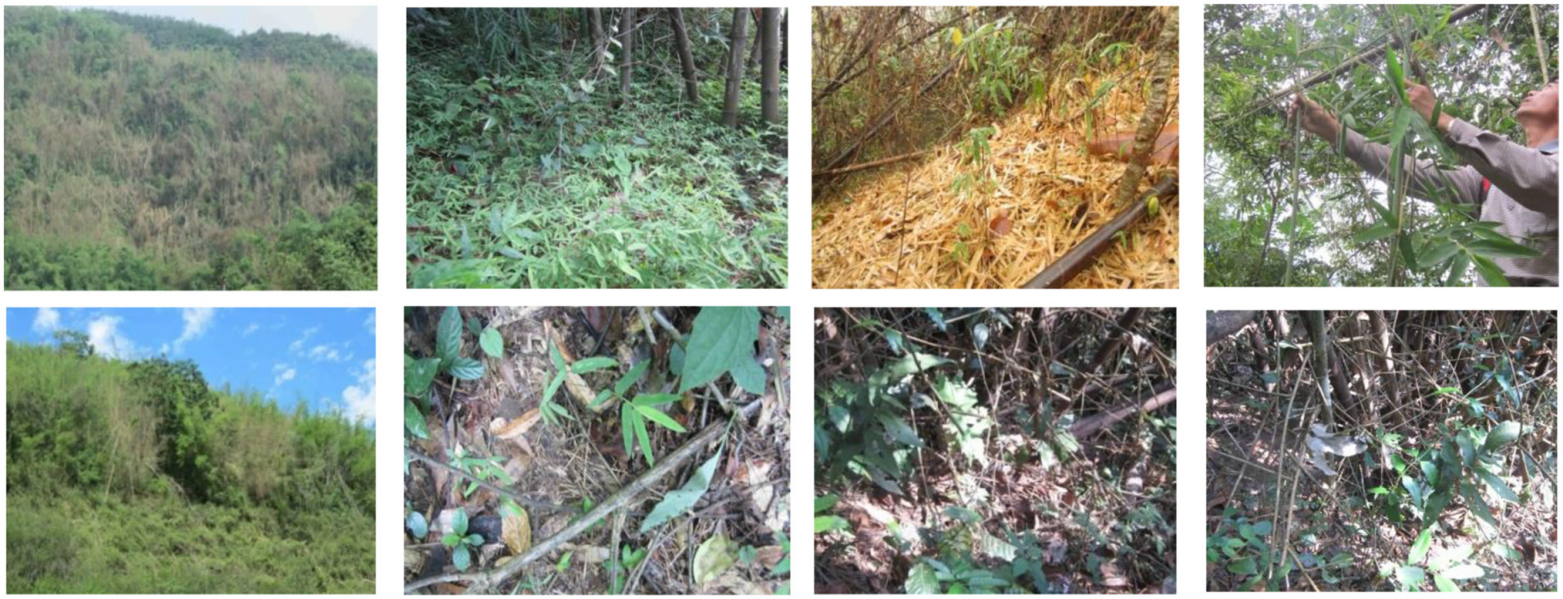
Mass flowering (upper row, quadrat A) and sporadic flowering (lower row, quadrat B) of *D*. *membranaceus* forests in southern Yunnan, China, and one-, two- and three-year-old seedlings.

A distinctive feature of most woody bamboo species is that flowering and seed set is followed by total death of all the culms, which might cause massive decline of the bamboo population and severe ecological change, especially if there is no significant natural regeneration [9,10,11]. However, due to the long period of vegetative growth and low seed set, natural regeneration after flowering and death are rarely observed and evaluated in the field [12,13]. Generally, there are two major types of bamboo flowering, i.e., sporadic and mass (gregarious) flowering, in the wild. Ecological observations of bamboo flowering have been rarely documented, such as for *Schizostachyum zollingeri* [14], *Sasa kurilensis* [15], *Dendrocalamus giganteus* [16]. In the case of *D*. *membranaceus*, little is known about the demographic aspects of the early regeneration process, which involves flowering, fruiting, seedling establishment and growth. Since 2008, many species of bamboo started to flower because of prolonged and severe drought in many areas of Yunnan. During the last five years, both mass and sporadic flowering of *D*. *membranaceus* were observed in natural populations in southern Yunnan, which offered a unique opportunity to examine aspects of its sexual reproduction and natural regeneration (Fig 1).

In this study, three study sites were selected in the central distribution area of *D*. *membranaceus* at the XNNR in Yunnan, China. We focused on seed set, germination characteristics and natural regeneration of the two forms of flowering from 2013 to 2015. To this end, we performed a detailed investigation of seedling survival and development among mass and sporadically flowering *D*. *membranaceus*. The objectives of the study were: (1) to quantify bamboo seedling emergence, mortality and growth; (2) to analyze the natural regeneration typical of the different flowering phenomena; and (3) to provide possible scientific insights for the conservation and natural forest management of *D*. *membranaceus*.

## Materials and Methods

### Ethics Statement

The permission for each location was issued by Jinghong Forestry Bureau. No specific permits were required for the described field studies involving the investigations of seed setting and germination, as well as growth and survival of seedlings from the studied bamboo forest. The field studies did not involve endangered or protected plant or animal species.

### Study site

**Fig 2:**
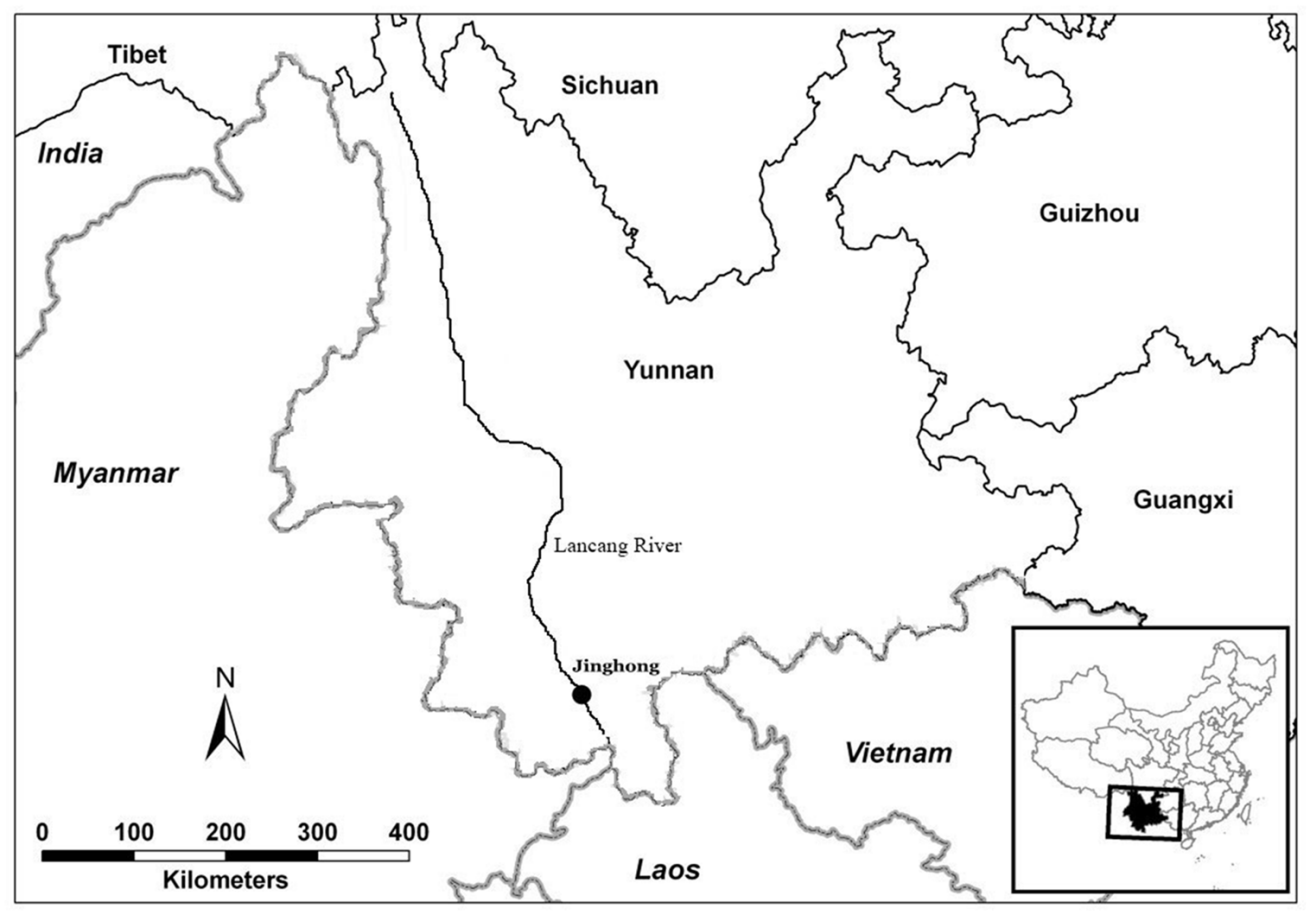
Location of the study site in Yunnan Province, China.

The investigations were conducted in *D*. *membranaceus*-dominated forest inside the XNNR in Jinghong County, Xishuangbanna Autonomous Prefecture, Yunnan, China (Fig 2). The XNNR is located at 21°48′-22°20′N, 100°36′-101°22′E, with an elevation range of 600-900m. The site is located downstream of the Lancang River where *D*. *membranaceus* is the dominant species in native forests relatively little disturbed by humans. The mass and sporadic flowering of *D*. *membranaceus* occurred from the winter of 2011 with their respective peaks in summer and autumn of 2012. We selected three fixed observation sites, A, B and C (Table 1, S1 Table), at the end of 2012. The mass flowering population was observed in quadrat A and sporadically flowering populations were studied in quadrats B and C. The quadrat A was in pure *D*. *membranaceus* forest, in which 52 clumps were flowering of a total of 61 clumps (85.2%). The total cover of herb layer (TCHL) was 0.4 and 0.5 in July and November 2013, respectively. The herb layer in quadrat A was mainly composed of weeds of Poaceae and ferns, such as *Microstegium ciliatum* (Poaceae) and *Cyclosorus molliusculus* (Thelypteridaceae). The quadrat B is forest of mixed bamboo and tree vegetation (bamboo: tree = 2:8). There were five flowering clumps among 38 bamboo clumps (13.2%) in quadrat B. The TCHL was 0.6 and 1.0 in July and November 2013, respectively, and the herb layer was mainly dominated by weeds of Zingiberaceae and Asteraceae with big or broad leaves, such as *Curcuma longa* (Zingiberaceae), *Eupatorium adenophorum* (Asteraceae), as well as the fern *Lygodium polystachyum* (Lygodiaceae) and the legume *Flemingia macrophylla* (Leguminosae). The quadrat C is also bamboo-tree mixed forest (bamboo: tree = 9:1). In quadrat C, eight flowering clumps were found among 55 bamboo clumps (14.5%). The TCHL was 0.5 and 0.9 in July and November 2013, respectively, and the herb layer was similar to that in quadrat B, mainly dominated by *Curcuma longa*, *Geophila herbacea* (Rubiaceae), *Lepidagathis incurve* (Acanthaceae) and *Eupatorium adenophorum* (Asteraceae). All three quadrats are located on the north bank of the Mengyang River, are close together and have very similar soil and light conditions throughout [7].

**Table 1:** Study sites for seed set and natural regeneration of D. membranaceus in Yunnan, China.

Location	Quadrat area (ha)	Longitude	Latitude	Elevation (m)	Flowering type and number of flowering bamboo groves
A: Pure *D*. *membranaceus* forest	2.00	100°52′28″	22°01′52″	753.4	Mass flowering, 52 clumps flowering among 61 clumps, 4 clumps were randomly selected for regeneration observation. Distances between flowering clumps sampled are 10–25 m.
B: bamboo-tree mixed forest (bamboo: tree = 2:8)	0.67	100°52′05″	22°10′23″	821.5	Sporadic flowering, 5 clumps flowering among 38 clumps, all 5 flowering clumps were observed for regeneration. Distances between flowering clumps are 10–20 m.
C:bamboo-tree mixed forest (bamboo: tree = 9:1)	1.00	100°52′32″	22°09′50″	810.0	Sporadic flowering, 8 clumps flowering among 55 clumps, all 8 flowering clumps were observed for regeneration. Distances between flowering clumps are 6–12 m.

The XNNR has a typical tropical monsoon climate. Annual temperature averages 21.3°C; and sunshine duration is between 1,820 and 2,179 h. Annual precipitation ranges from 1,207 to 1,533 mm and approximately 85% of the total precipitation occurs in May to October. There is a distinct rainy season (from May to October) and dry season (from November to next April) in Xishuangbanna, including the XNNR [2,3,6,17]. Commonly, leaves of *D*. *membranaceus* senesce yellow and fall in the dry season and then new leaves grow in the rainy season in Yunnan, China [2,3].

### Seed set

To examine the seed set in mass and sporadic flowering populations, the numbers of spikelets and seeds were counted for three 1-meter-long flowering branches collected from each of the upper, middle and lower parts of flowering culms (i.e., nine branches in total) for each flowering clump. The measurements were conducted with three clumps in quadrat A, five clumps in quadrat B and eight clumps in quadrat C (S2 Table). Although *D*. *membranaceus* spikelets can have 2–4 flowers, most spikelets normally bear only one seed, rarely two or more seeds. The seed set per spikelet is estimated by the following formula: seed set (%) = total number of seeds /number of total spikelets × 100.

### Seed germination

Comparative observations of seed germination and seedling growth between the different flowering populations were carried out to evaluate the extent of natural regeneration in quadrats A, B and C from January 2013 to November 2015.

The germination rates of seeds collected from the mass and sporadically flowering populations were investigated in the nursery. For each quadrat, seeds from all investigated flowering clumps were mixed before germination experiments were conducted. After sterilizing and washing, three replicates of 50 seeds were sown and placed in a chamber with constant temperature of 25°C and under a 12 h light regime. Then germination was monitored at 2-day intervals for 30 days. Radicle emergence within three weeks after sowing was taken as a sign of successful germination.

### Clump shadow and seedling counts

We measured the "clump shadow" as the ground area defined by the outermost reach of all culms in the clump and excluding the basal area occupied by the culms. Seedling counts were divided by the clump shadow to obtain the seedling densities within each quadrat.

### Growth and survival of seedlings

Seed germination, seedling growth and survival were observed at the early part, middle and end of each year. Seedling growth was determined by measuring the diameter at ground level and height of the largest emerged culm of seedlings.

### Statistical analysis

SPSS 17.0 software was employed for statistical analysis and least-significant difference (LSD) multiple comparisons.

## Results

### Seed set during mass and sporadic flowering

In general, the seed set in natural populations of *D*. *membranaceus* was low, ranging from 1.76% to 7.49% (Table 2). The seed set in quadrat A of the mass flowering population (7.49% ± 0.40) was significantly higher (4.26 and 3.67 times) than those in quadrats B and C of the sporadically flowering population (1.76% ± 0.52 and 2.04% ± 0.22, respectively). The seed set values in quadrats B and C of the sporadically flowering population did not differ significantly (Table 2).

**Table 2:** Comparison of seed set and germination rates of mass flowering (A) and sporadic flowering (B, C).

Quadrat	Seed set (%)	Seed germination		
		Germination rate (%)	Time to first germination (d)	Total germination period (d)
A	7.49 ± 0.40a	71.0 ± 5.5a	7±1	15±1
B	1.76 ± 0.52b	63.4 ± 6.7b	5±2	13±2
C	2.04 ± 0.22b	59.6 ± 5.7b	7±1	10±3

Note: Values are mean ± standard deviations. The same letters after germination rates indicate lack of statistical significance between treatments, and different letters represent statistically significant difference at p<0.05.

### Seed germination

Germination of fresh seeds of *D*. *membranaceus* began just 5–7 days after sowing and lasted 10–15 days. The germination rates of seed from the three quadrats ranged from 59.6% to 71.0% (Table 2). The seed germination from the mass flowering population (quadrat A) was 71.0± 5.5%, 1.12 and 1.19 times significantly higher than those in quadrats B and C of the sporadically flowering population (63.4 ± 6.7% and 59.6 ± 5.7%, respectively). There was no significant difference in germination rate between quadrats B and C.

### Seed dispersal, seedling establishment and survival

The bamboo clumps of the three quadrats began flowering and fruiting from November 2012 to May 2013, lasting ca. 6 months. Seed germination was observed through ca. 7 months, from January to July of 2013, and germination was concentrated in the rainy season, i.e., May to October. The seeds of flowering bamboo clumps mainly fell within their "clump shadow", so the seedlings were also distributed likewise (Table 3). The highest number and density of seedlings averaged for a single clump was observed in July 2013, 2249.13 plants per clump and 36.66 seedlings/m^2^, 470.53 plants per clump and 10.66 seedlings/m^2^, and 77.25 plants per clump and 3.40 seedlings/m^2^ in quadrats A, B and C, respectively. The final number and density of seedlings are significantly less than in the beginning, which might be attributed to various factors including different bamboo clump sizes, and differences in the development of the herb layer around the clump, which competes against bamboo seedlings.

**Table 3:** Average number and density of D. membranaceus seedlings in the mass flowering (A) and sporadically flowering (B, C) quadrats.

Quadrat	Average diameter of clump shadow (m)	Average number (seedlings/clump) and density (seedlings/m^2^) of seedlings in the clump shadow.								
		2013			2014			2015		
		January	July	November	March	August	November	March	August	November
A	8.83a	462.13a/7.53a	2249.13a/36.66a	1328.72a/21.66a	788.89a/12.86a	470.88a/7.68a	308.44/5.03	49.69/0.81	12.75/0.21	3.42/0.06
B	7.50b	69.91b/1.47b	470.53b/10.66b	118.34b/2.68b	14.13b/0.32b	0.80b/0.02b	--	--	--	--
C	5.37c	5.23c/0.23c	77.25c/3.40c	9.85c/0.43c	0.50 c /0.02c	--	--	--	--	--

Notes:--indicates that all seedlings were dead.

We were able to compare seedling survival for all three quadrats during a window of just under a year, between July 2013 (when seedling numbers peaked) and March 2014 (the month after which we do not have complete data for all three quadrats; see Table 3). Basically, the status of seedling survival in all three quadrats showed the same trend, i.e., high mortality and a sharp drop in the number of seedlings (survivorship). Compared to July 2013, both the number and density of seedlings in quadrat A had declined by 64.92% by March 2014; meanwhile the decline was 97.00% and 99.35% in quadrats B and C, respectively. All seedlings and parent clumps in quadrats B and C were dead within two years, i.e., from 2013 to 2014, indicating unsuccessful natural regeneration within the sporadically flowering populations. Remarkably, a considerable number of seedlings in quadrat A survived after three years. By November 2014, although both the number and density of seedlings in quadrat A had declined by 98.89%, there was still an average of 3.42 seedlings per clump that survived; the density of seedlings was 0.06 seedlings/m^2^ in November 2015 (Table 3).

### Height and culm diameter development of seedlings

Generally, seedling height and diameter at ground level in all three quadrats kept increasing during 2013. However, the increments in culm height and diameter at ground level in quadrat A seedlings were much higher than those in quadrats B and C (Table 4). Between January and November 2013, the average seedling height increased by 9.74, 2.79 and 1.29 times in quadrats A, B and C, respectively, and the average diameter at ground level increased by 1.79, 0.63 and 0.74 times, respectively. Beyond the first year from January 2013 to March 2014, the height of seedlings in quadrats A, B and C had increased even more, by 1053.25%, 289.69% and 155.19%, respectively; similarly, the diameter at ground level of seedlings had increased 410.71%, 76.32% and 88.89%, respectively. On the other hand, the results appear significantly different for 2013 and 2014. Between March and the rainy month of August 2014, the average height and diameter for quadrat A had increased by 73.02% and 146.85%, respectively, while these attributes in quadrat B only increased slightly by 14.59% and 2.99%, and all the seedlings in quadrat C were dead. By 2015, the only surviving seedlings were in quadrat A. From March to November 2015, the average height and diameter had further increased tremendously, by 321.37% and 147.80%, respectively (Table 4).

**Table 4:** Culm height and diameter at ground level of seedlings compared for the mass flowering (A) and sporadically flowering (B, C) quadrats.

Quadrat	Average height(cm) and diameter at ground level (mm) of largest seedling culms								
	2013			2014			2015		
	January	July	November	March	August	November	March	August	November
A	3.23a/0.28a	17.15a/0.45a	34.70a/0.78a	37.25a/1.43a	65.45a/3.53a	75.28/4.03	88.32/4.54	237.26/9.38	372.15/11.25
B	2.62b/0.38b	6.84b/0.60b	9.97b/0.62b	10.21b/0.67b	11.70a/0.69b	--	--	--	--
C	2.70b/0.27a	5.98b/0.37a	6.17c/0.47c	6.89c/0.51b	--	--	--	--	--

Average height and diameter increments in the three quadrats appeared to be significantly different from 2013 to 2015. This might be attributed to few or no shoots produced at quadrats B and C even during the rainy season in 2014 and 2015. Furthermore, even if a small number of tillers appeared on the nodes of culms, only seedling height increased and diameter values remained unchanged. In contrast, in the mass flowering population represented by quadrat A, new culm shoots arose from the seedling rhizomes during the rainy seasons of 2014 and 2015, so that height and diameter values increased yearly from 2013 to 2015, indicating a positive tendency to natural regeneration.

## Discussion

### Seed set and germination rate between mass and sporadically flowering populations

In this study, the seed set in natural populations of *D*. *membranaceus* in Yunnan, China, was found to be considerably low, less than 10%. Notwithstanding, the seed set in mass flowering populations was significantly higher than that of sporadically flowering populations. Mizuki et al. [18] also found that seed set increased with flowering area in *Sasa* species. Such results may be attributable to the typical wind-pollination characteristic among woody bamboos [12,19,20] and short pollen viability [21]. In *D*. *membranaceus*, the massive synchronized flowering could supply more available pollen than sporadic flowering events, and so could improve seed set [9,12]. Additionally, more bees (such as *Apis cerana* Fabricius) were observed to visit the fresh inflorescences of mass-flowering *D*. *membranaceus* populations on sunny mornings and afternoons, which could also enhance pollination success and seed set. Likewise, Wong [14] also observed bees foraging within a gregariously flowering stand of *Schizostachyum zollingeri* bamboo. Such bees were clearly exploiting an increased availability of pollen in their territory.

As shown in previous studies [22], the germination rates of fresh seeds of *D*. *membranaceus* were reasonably high. Futhermore, the nursery germination rate of seed material from the mass flowering population was also significantly higher than that from the sporadically flowering populations. A possible explanation is that in the mass flowering population, a greater number of potential male and female parents (genotypes) was available that improved outcrossing pollination rates with resulting heterosis in the offspring [23]. On the other hand, for the sporadically flowering populations, fewer flowering individuals might aggrandize self-pollination rates, possibly causing increased homozygosity in offspring, leading to deleterious allele accumulation and inbreeding depression [24]; such inbreeding depression will be expressed post-zygotically as reduced seed germination in bamboo [19,25].

Our results indicate that seeds of *D*. *membranaceus* had no dormancy and could germinate in 5–7 days following ripeness, as with other tropical and subtropical woody bamboos [14,22]. For successful natural regeneration, rapid seed germination has its advantages. Lack of seed dormancy, high germination rate and a short germination period in *D*. *membranaceus* will be conducive to quick natural regeneration, as a counter-balance to potentially vacating the ecological niche following mass flowering and whole-clump senescence and death [26,27]. However, environmental factors, such as light quality, water, temperature and soil nutrient conditions, can influence bamboo seed germination and seedling growth [27,28].

### Comparison of natural regeneration in mass and sporadically flowering populations

Studies of seedling establishment and survival in mass flowering monocarpic temperate bamboos have shown healthy regeneration that were attributable to high seed set and increased heterosis [15,28]. Our results comparing mass flowering and sporadically flowering *D*. *membranaceus* populations have also reinforced this possibility in tropical woody bamboos. Compared with sporadically flowering populations, clumps in the mass flowering population produced more seed with higher germination rates, resulting in a much greater number of seedlings. Despite high seedling mortality over the subsequent two years (64.92% in 2014 and 98.89% in 2015), the greater seedling survival indicated better regeneration potential in the mass flowering population. *D*. *membranaceus* also has a strong asexual reproductive capacity, and even only one surviving underground stem can develop into a lush new clump with enough precipitation [2]. Given the relatively high survival rate of seedlings in quadrat A, as well as increasing precipitation since the rainy seasons of 2013 in Jinghong County, it would be reasonable to speculate good natural regeneration. What is more important is the growth vigor of most seedlings in the mass flowering population which is indicated by increased shoot growth in the rain season. This could be the signal that the seedling population has entered a more density-stable phase in the early regeneration of *D*. *membranaceus* [15]. In contrast, most seedlings from the sporadic flowering were unable to produce shoots from their rhizomes and all seedlings were dead in the next two years, so that natural regeneration was unsuccessful.

The different seedling regeneration profiles of the two types of flowering *D*. *membranaceus* populations were probably also influenced by local ecological conditions. The precipitation during and after the rainy seasons of 2013, in Jinghong County where these studies were based, promoted not only the seed germination of *D*. *membranaceus* but also the growth of weedy understory shrubs and herbs. Moreover, the herb layer grew faster with the rains, as apparent from their overtopping the *D*. *membranaceus* seedlings. For the mass flowering population, the ground was covered with a 3~8 cm thick layer of leaf litter, resulting from a greater density of senescent and defoliating clumps, that could retard both bamboo and other plant establishment due to germinating radicles not easily finding contact with mineral soil. In such circumstances, the amount of seed rain and smaller size of propagules (in this case fallen inflorescence units called pseudospikelets containing the seed or seeds from the bamboo that could be easily washed under the litter layer) could conceivably result in a more competitive establishment than other plants on site. This is reflected by the herb cover that was little changed in quadrat A, from 0.4 in July to 0.5 in November after the rainy season of 2013. Also, this leaf litter layer might provide better soil moisture retention that benefits seedlings of *D*. *membranaceus*. On the other hand, *D*. *membranaceus* in the sporadic flowering populations was mixed with broad leaved trees, and from 2013 to 2015, the TCHL in the sample plots increased much from 0.5~0.6 in July, to 0.9~1.0 in November, including many herbs with large leaves. Overtopping by such a herb layer is extremely disadvantageous to growth of *D*. *membranaceus* seedlings. Shading by the herb layer conceivably also contributes to bamboo seedling deterioration or death on site.

## Conclusion

*D*. *membranaceus* forest is one of the most important vegetation covers for soil and water conservation in parts of southwest China, including the Lancang River Valley. In recent years, as a result of deforestation and land use changes, the natural area of *D*. *membranaceus* forest has declined sharply, and has even resulted in a dwindling of water resources, desertification and a degraded ecological environment where such forest was removed. Due to long and severe droughts during 2008 to 2012, *D*. *membranaceus* stands began to flower frequently and subsequently declined. An effective conservation and management approach for a key resource such as this is urgently needed. Our present results contribute some useful insights towards the protection and management of natural *D*. *membranaceus* forests.

Seeds are one of the most convenient germplasm resources for plant conservation and the frequent flowering of *D*. *membranaceus* has offered opportunities for collecting material from different genetic provenances of this species, an important management goal. In view of significant differences in seed set, natural regeneration ability and sustainability of bamboo populations between the mass and sporadically flowering populations, different management measures might be considered. For the sporadically flowering populations, the poor natural regeneration and mortality of parent bamboo clumps after flowering suggest that to maintain a mixed *D*. *membranaceus* forest on site, other provenances of this species might be introduced to site, so as to increase the genetic diversity of the bamboo population. On the other hand, the mass flowering population is able to regenerate naturally, so that protective measures can be taken, such as closing the land for reforestation and prohibiting human intrusion, to allow natural recovery processes, which would be one of the most economical approaches.

## Supporting Information

S1 Table Locations and summary data of three quadrats. (PDF)

S2 Table Number of bamboo clump samples for observation and measurements. (PDF)
