# Supplementary material for: Seed Set and Natural Regeneration of Dendrocalamus membranaceus Munro after Mass and Sporadic Flowering in Yunnan, China
Source: PLoS One. 2016 Apr 14;11(4):e0153845. doi: 10.1371/journal.pone.0153845 (PMC4831783; doi:10.1371/journal.pone.0153845)
Supplement: S1 Table — (PDF) [file pone.0153845.s001.pdf]

S1 Table. Locations and summary data of three quadrats.

Quadrat	Area (ha)	Location			Number of bamboo (clump)		
		Longitude	Latitude	Elevation (m)	Total	flowering	sample (observed)
A:	2.00	100°52′28″	22°01′52″	753.4	61	52	4
B:	0.67	100°52′05″	22°10′23″	821.5	38	5	5
C:	1.00	100°52′32″	22°09′50″	810.0	55	8	8
