# Supplementary material for: Seed Set and Natural Regeneration of Dendrocalamus membranaceus Munro after Mass and Sporadic Flowering in Yunnan, China
Source: PLoS One. 2016 Apr 14;11(4):e0153845. doi: 10.1371/journal.pone.0153845 (PMC4831783; doi:10.1371/journal.pone.0153845)
Supplement: S2 Table — (PDF) [file pone.0153845.s002.pdf]

S2 Table. Number of bamboo clump samples for observation and measurements (clump).

Quadrat	Rate of seed set	Seedling development		Regeneration
		Growth	Survival	
A	3	4	4	4
B	5	5	5	5
C	8	8	8	8
